# Marine Bioactive Peptides in the Regulation of Inflammatory Responses: Current Trends and Future Directions

**DOI:** 10.3390/proteomes13040053

**Published:** 2025-10-13

**Authors:** D. M. N. M. Gunasekara, H. D. T. U. Wijerathne, Lei Wang, Hyun-Soo Kim, K. K. A. Sanjeewa

**Affiliations:** 1 Department of Biosystems Technology, Faculty of Technology, University of Sri Jayewardenepura, Homagama 10200, Sri Lanka; nimeshamg2@gmail.com; 2 Department of Seafood Science and Technology, Institute of Marine Industry, Gyongsang National University, 2–9, Tongyeonghaean-ro, Tongyeong-si 53064, Gyeongsangnam-do, Republic of Korea; tudayangani14@gmail.com; 3 State Key Laboratory of Marine Food Processing & Safety Control, College of Food Science and Engineering, Ocean University of China, Qingdao 266003, China; leiwang2021@ouc.edu.cn; 4 Sanya Oceanographic Institution, Ocean University of China, Sanya 572024, China

**Keywords:** marine bioactive peptides, inflammation, marine bioresources, pro-inflammatory cytokines

## Abstract

Marine-derived bioactive peptides (MBPs) are emerging as promising natural agents for regulating inflammatory responses. MBPs, typically obtained through enzymatic hydrolysis of proteins from various marine organisms such as fish, mollusks, and algae, exhibit diverse biological activities, including antioxidant, immunomodulatory, and anti-inflammatory effects. The ability of MBPs to modulate key inflammatory mediators such as TNF-α, IL-6, and COX-2, primarily through pathways like NF-κB and MAPK, highlights the therapeutic potential of MBPs in managing chronic inflammatory diseases. However, most existing studies are confined to in vitro assays or animal models, with limited translation to human clinical applications. This review explores the stability, bioavailability, and metabolic rate of MBPs under physiological conditions, which remain poorly understood. In addition, a lack of standardized protocols for peptide extraction, purification, and efficacy evaluation hinders comparative analysis across studies and also different proteomics approaches for separation, purification, identification, and quantification of marine-derived peptides with therapeutic properties. The structure–function relationship of MBPs is also underexplored, limiting rational design and targeted applications in functional foods or therapeutic products. These limitations are largely due to a lack of consolidated information and integrated research efforts. To address these challenges, this review summarizes recent progress in identifying MBPs with anti-inflammatory potentials, outlines key mechanisms, and highlights current limitations. Additionally, this review also emphasizes the need to enhance mechanistic understanding, optimize delivery strategies, and advance clinical validation to fully realize the therapeutic potential of MBPs.

## 1. Introduction

Inflammation is the body’s first immune response to foreign substances such as toxins, allergens, pathogens, injury, or damaged tissue. It involves complex interactions between blood vessels, immune cells, and molecular and cellular mediators [1,2,3,4]. This response aims to restore normal body function by eliminating the initial cause of injury and removing damaged cells by producing pro-inflammatory enzymes and cytokines such as inducible nitric oxide synthase (iNOS), cyclooxygenase (COX-2), tumor necrosis factor (TNF-α), and interleukins (IL-1β) (IL-6), primarily through several pathways, including nuclear factor-kappa B (NF-κB), mitogen-activated protein kinases (MAPKs), janus kinase (JAK)/signal transducer and activator of transcription 3 (STAT3), and NOD-like receptor family pyrin domain containing 3 (NLRP3) [5,6]. This inflammatory process plays a significant role in wound healing and microbial resistance [7]. However, when inflammation becomes dysregulated and prolonged, it can lead to different chronic diseases and significant threats to individual health and longevity [8]. In the past decade, inflammation has been identified as a silent global issue due to long-term inflammation underlying several human diseases, such as cancer, diabetes, arthritis, neurodegeneration, cardiovascular disease, and inflammatory bowel disease (IBD) [9]. To alleviate or prevent inflammatory diseases, several therapeutic strategies, including corticosteroid medications, nonsteroidal anti-inflammatory drugs (NSAIDs), biological agents, and lifestyle modifications, are implemented [10]. Despite that, several chronic inflammatory disorders have no fully effective cure and are primarily managed through symptomatic therapies, while common existing drugs show considerable side effects with long-term use [11]. For instance, currently available drugs, aspirin and indomethacin, which are NSAIDs, often help reduce pain caused by inflammation. However, despite their therapeutic effectiveness, they are also associated with potential adverse reactions, mostly stomach ulcers, and rarely, stroke and myocardial infarction [12]. Therefore, recent studies have focused on searching for novel anti-inflammatory drugs from natural bioresources to overcome limitations present in synthetic medications.

The marine ecosystem provides a rich habitat for immense biodiversity, encompassing a diverse range of habitats from highly productive coastal regions to deep-sea environments. This wide range of distribution supports a varied series of life on the planet, allowing for extraordinary interactions among marine plants, animals, and microorganisms, forming complex food webs and contributing to global biogeographical cycles, such as carbon, nitrogen, and oxygen [11]. Consequently, the ocean maintains marine ecosystems and the ecological balance by stabilizing the sustainable biosphere. To date, humans are the most advantageous users of marine resources that extend beyond food and medicines. Therefore, further comprehensive explorations of marine biodiversity are essential for developing new scientific uses [13,14]. Extreme maritime conditions, such as salinity, temperature, pressure, illumination, and variable oxygen levels, produce unique and diverse compounds when compared to the terrestrial environment [3]. The adaptation of these conditions has been directed to the production of by-products, including proteins and bioactive peptides, lipids, polysaccharides, phenolic compounds, and pigments with distinct structural features, diversity, and functional properties [15]. This variety of natural bioactivities has become a more sustainable solution for various industries, especially in medicine, cosmetics, and food, which is due to their abundance, low toxicity, and high specificity. Among them, the medical industry has become more exploratory in research on natural compounds because synthetic compounds have become less effective and are often highly toxic [16]. Marine bioactive compounds exhibit diverse biological activities, including antioxidant, anti-diabetic, anticancer, antimicrobial, immunomodulatory, angiotensin-converting enzyme (ACE) inhibitory activity, and anti-inflammatory effects, and many other biomarkers which have recently been implicated in human health [17]. To date, marine bioactive compounds are directly or indirectly responsible for approximately 60% of pharmaceutical bioactivities as alternative and or complementary therapies [18]. According to the recently reported data, about 68% of cancer-treated drugs are derived from marine organisms, while the remaining are utilized for inflammation, pain, and other medical conditions [19]. Proteomics has been identified as an essential approach for identifying anti-inflammatory targets and assessing therapeutic responses associated with inflammatory diseases. Rather than proteomics, peptidomics has become an emerging and novel field that improves efforts in peptide drug discovery [20]. However, most current research remains confined to in vitro assays or animal models, with minimal translation to human clinical applications. Aquatic proteomics is one of the advancing analytical technologies, useful for studying the dynamic changes within the proteome [21]. It enables detailed insights into protein-related processes, such as protein synthesis, post-translational modifications (PTMs), and degradation, in response to diverse marine environmental factors and developmental (ontogenetic) stages in marine organisms [22].

**Figure 1:**
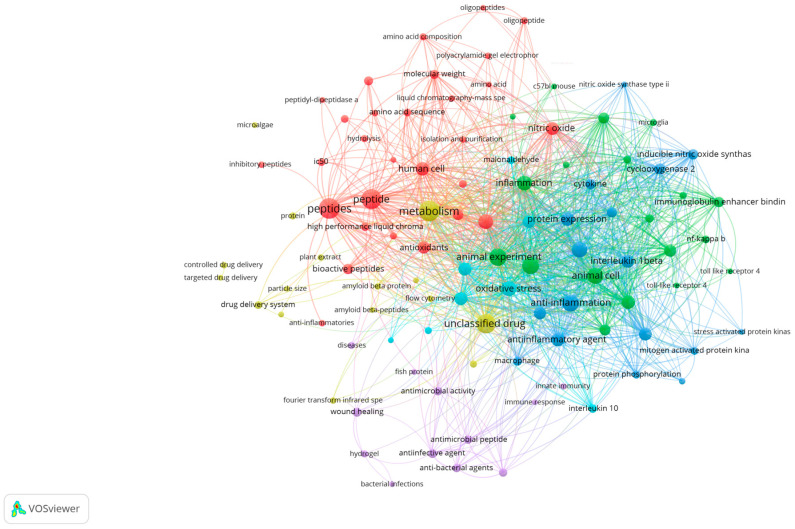
Bibliometric network analysis of recent publications (2020–2025) using VOSviewer (version 1.6.20), based on Scopus data. Clusters reveal thematic areas most relevant to marine bioactive peptides and inflammation.

This review aims to present an overview of research trends on MBPs, mainly focusing on their anti-inflammatory potential and the molecular mechanisms reported over the years 2020–2025. Additionally, the advantages and drawbacks associated with marine peptide separation, purification, identification, and quantification techniques are discussed, with an emphasis on current scientific knowledge and future perspectives in the pharmaceutical industry. The Scopus database and Google Scholar were searched to screen relevant articles in this review article. Articles reviewed in the results and discussion sections were based on novelty, time of publication, and the number of citations. For this study, a bibliographic analysis was conducted covering the studies published during 2020–2025, scanning the keywords MBPs, inflammation, marine bioresources, and pro-inflammatory cytokines to locate anti-inflammatory properties of marine bioactive compounds (Figure 1).

### Marine Bioactive Peptides (MBPs)

Peptides are an intermediate product of protein synthesis, formed by amino acids connected by peptide bonds [23]. Bioactive peptides (BPs) are a type of natural compound consisting of specific protein fragments made up of 3 to 30 amino acid residues. BPs are inactive when integrated within proteins and are only biologically active when disrupted during fermentation, processing, or gastrointestinal digestion [24]. The structure can be linear or cyclic and may contain secondary structures like turns and loops, and exhibit various functional activities, mainly dependent on the sequence and composition of amino acids [25]. Normally, peptides exist as low-molecular-weight compounds of less than 2500 Da, thereby providing a greater possibility of passing the intestinal barriers free of restriction [26]. Peptides can be sourced from natural, synthetic, or recombinant origins [27]. Natural protein sources are diverse, including terrestrial plants, animals, and microorganisms, and each has advantages as well as serious limitations. The reasons for conformational instability, a short half-life, and narrow biological activities have shifted the research attention towards marine-derived resources [28]. Recently, extensive research on marine bioresources has been attributed to their diverse biological properties, as well as their functional properties, such as solubility, emulsification, and gel formation properties, which can be beneficial for industrial applications [24]. At present, exploration of marine bioactive products (MBPs) has reached more than 40,000 compounds, and each year, over 1000 secondary metabolites are discovered, the majority of which exhibit anticancer and cytotoxic effects [29,30].

**Figure 2:**
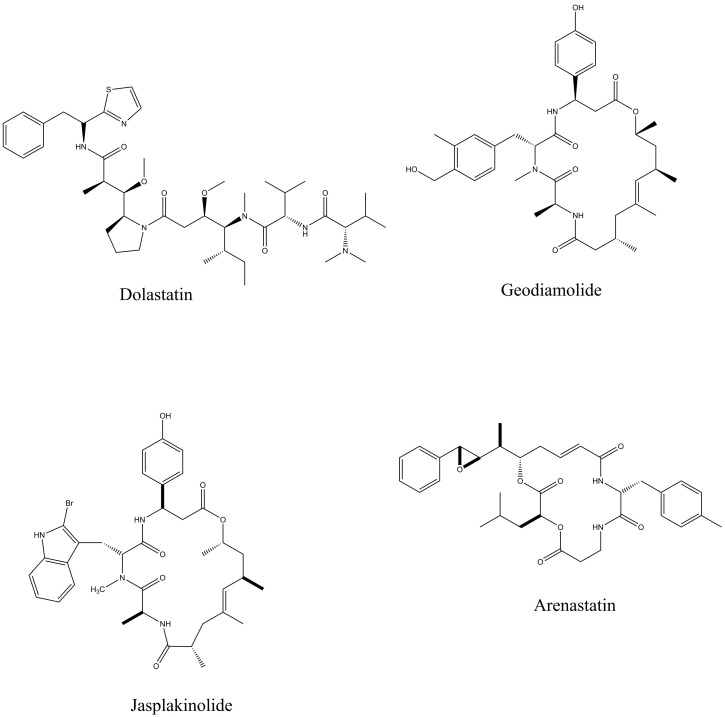
Bioactive peptides from seaweeds, sponges, mollusks, and ascidians that exhibit therapeutic effects.

**Figure 3:**
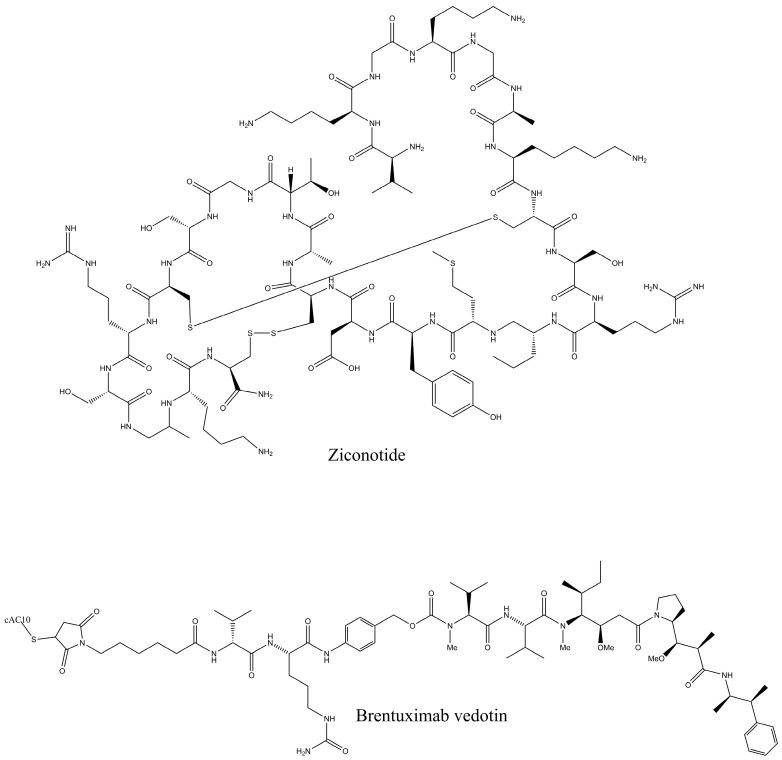
FDA-approved marine peptide-based compounds.

Peptides have been obtained from vast marine sources, including algae (macro and micro), ascidians, sponges, fish, bivalves, mollusks, crustaceans, and some marine by-products, such as shellfish, fish skins, viscera, and muscles. Peptides are highly enriched with multiple pharmacological activities, including anticancer, antimicrobial, antioxidant, anti-diabetic, anticoagulant, anti-inflammatory, hepatoprotective, cardioprotective, immunomodulatory, neurogenerative, and appetite-suppressing effects, and many more [1,24]. Among them, extracted bioactive peptides from seaweeds, sponges, mollusks, and ascidians have shown greater application in pharmaceutical properties (Figure 2) [31]. Moreover, 71.74% of collagen hydrolysate peptides, which contain glycine, proline, hydroxylysine, or hydroxyproline, primarily derived from marine fish skin, scales, and bones, have demonstrated significant potential in preventing and managing various health conditions [32]. Considering the safety aspects of MBPs is essential for pharmaceutical products. In comparison to numerous other bioactive compounds, MBPs are comparatively less allergic and exhibit minimal or no toxicity. However, risk factors such as heavy metals, high iodine levels, anti-nutrients, pesticide residues, ammonia, and radioactive substances may coexist with the final product if the production process fails to implement appropriate safety measures [33]. A number of MBP-based pharmaceutical products have reached the market, while most of the drugs are in preclinical and clinical stages. Nevertheless, some of these products may later be rejected or withdrawn due to emerging safety concerns and limited efficacy. Peptides are the fundamental components of proteins and are essential for both the structure and function of the proteome, providing valuable insights into the dynamic nature and complexity of the cellular proteome. Proteomics approaches have involved unveiling how marine life adapts to its environment and identifying biologically active proteins or peptides that could have mainly therapeutic applications, particularly those that use peptide analysis. Primarily, three techniques, solvent extraction, chemical and enzymatic hydrolysis, and microbial fermentation, are used for releasing peptides from different marine sources. The enzymatic hydrolysis technique is the most common for peptide isolation, but some studies for specific peptides have shown that chemical synthesis and recombinant DNA technologies are more effective for large-scale production. Further, the study performed by Sridhar, Inbaraj et al., 2021 has summarized some more advanced technologies for isolation, purification, and identification, which have been used more recently by suggesting that a combination of different processing technologies can be used for enhancing the yield and purity of the MBPs [34]. To date, Ziconotide (Prialt^®^) has been recognized as the first marine-derived peptide approved by the Food and Drug Administration (FDA) in 2004 [35]. This drug is isolated from the marine cone snail, *Conus magus*, which is used to relieve chronic pain by blocking specific voltage-activated calcium channels in neurons [36]. Furthermore, Brentuximab Vedotin (Adcetris^®^), isolated from the marine mollusk, *Dolabella auricularia*, was approved in 2011 [36]. This is an antibody–drug conjugate designed to target CD30-positive cells, making it effective in the treatment of classical Hodgkin lymphoma and systemic anaplastic large cell lymphoma [Figure 3].

Further, Jin, Peng et al., 2022 highlighted biological activities, mainly anticancer, antimicrobial, and antioxidant ones, and their relationships with various marine organisms. According to the study, Dolastatin 10, a low-molecular-weight cyclic anticancer peptide, shows the greatest potential for medical use. The development of new Dolastatin 10 analogs has even led to their incorporation into modern conjugated drugs. The peptides Geodiamalide and theopederin A are the marine sponge-derived peptides that show strong cytotoxicity, and theopederin A has indicated the greatest antitumor activity [37].

Nevertheless, a considerable amount of research has been conducted on peptide-based drugs; the majority are in the preclinical stage, and only a small number have reached phase I, II, and/or III of clinical trials (Table 1). Surprisingly, various food and nutraceutical products are being made with ingredients derived from MBPs or peptide-rich hydrolysates of fish gelatin, collagen, and fish proteins. These products have the ability to treat or alleviate chronic diseases, especially for bone and intestinal health, reducing anxiety, controlling postprandial blood glucose levels, and providing overall nutritional supplements.

## 2. The Structure–Function Relationship of Marine Bioactive Peptides

The structure of MBPs determines their biological activities, and it is essential to analyze the relationship between their structures and the bioactivity of MBPs to develop and optimize therapeutic uses [23]. According to the isolated marine source, the structures of the peptides are complex and highly variable, especially due to the high taxonomic diversity [45]. The molecular weight, amino acid composition and sequence, spatial conformation, and other factors, such as hydrophobicity, balance between charge density, and polymer chain length of the peptides, are crucially impacted on the biological activities [46]. Most commonly, MBPs are small in size and require support to be absorbed more efficiently by the body. Most antihypertensive and antioxidant peptides have low molecular weights (MWs), typically around 1 kDa; likewise, low-MW peptides contribute to the ACE inhibitory effects by increasing the binding ability to the active site of the ACE (Table 2) [23]. Moreover, the amino acid composition and sequence of the MBPs significantly affect several biological activities, such as antioxidant, anticancer, antimicrobial, and ACE inhibition. The anti-inflammatory activity of short sequences (2–3 amino acids) of peptides is generally absorbed more easily and effectively. Surprisingly, HCRG1 and HCRG2, which are long peptides (up to 50 amino acids) derived from the sea anemone (*Heteractis crispa*), have been reported to have intact absorption ability through the intestine at the tissue level. However, when increasing the chain length of the peptide sequence, the potency of administration tends to be decreased [47,48]. Further, the cyclic structure of peptides is often associated with better passive cell membrane permeability in comparison to the linear structures. Stylissatin A is a cyclic heptapeptide isolated from the marine sponge *Stylissa massa* that has shown anti-inflammatory effects, facilitating the reduction of nitrogen oxide (NO) production in lipopolysaccharide (LPS)-stimulated murine RAW264.7 macrophage cells (EC50 = 87 and 73 µM) [49]. A number of studies have confirmed that the variation in the type of the sequence and position of the amino acid and chemical properties, such as hydrophobicity, flexibility, and charged residues of the linear structure, along with their secondary and tertiary structure properties for the structure, enhances the functions of MBPs (Table 2). The positively charged amino acids in peptides act as chemokines and interact with the immune system, helping to suppress inflammation. It is reported that peptides derived from tuna juice have two positively charged amino acids, lysine (K) and arginine (R), most abundant at the N-terminal, and have anti-inflammatory activities [47].

Further, Rivera-Jiménez et al. (2022) reported that, after analyzing 31 peptide sequences, 19 contained between 25% and 100% hydrophobic amino acids, most commonly alanine and isoleucine. The tripeptide PAY, which is derived from salmon, shows 100% hydrophobicity and displays inhibitory activity against NO production [47]. The special conformation (3D structure) of MBPs largely determines their overall efficacy by enhancing their target-binding affinity through changing conformational structure. As a result, the activity will last longer by resisting degradation and preventing the loss of molecular stability.

## 3. Protein Hydrolysis Mechanisms (Protein Hydrolysis, Purification, Separation, and Identification)

### 3.1. Protein Extraction Process

Marine protein extraction involves isolating proteins from different marine sources. To date, enzymatic extraction, ultrasound-assisted extraction, deep eutectic solvent extraction, physical aided extraction, supercritical fluid extraction, and acidic extraction are commonly used. Depending on the species, amino acid composition, and sequence, extraction methods can vary [57]. For instance, microalgae are microscopic organisms and develop in various ecological environments. Spirulina (*Arthrospira platensis*) is a multicellular blue-green alga that belongs to the *cyanobacteria*, which contain a 55–70% protein content. Chlorella (*Chlorella vulgaris*) belongs to the phylum *Chlorophyta* and contains 42–58% protein. Using one-pot ultrasound-assisted extraction (UAE) gives a high yield of protein content simply, effectively, and sustainably [58]. *Chlorella vulgaris*, *Nanochloropsis oceanica*, and *Tetraselmis chuli* are other microalgae that contain high protein content. Researchers have gained high extraction yield using the freeze–thawing method and high-pressure homogenization from those microalgal species [59]. Collagen is one of the most abundant proteins found in animal bone and skin (pig, cow, and fish), and gelatin is the high-protein product derived from collagen [60,61]. Normally, enzymatic extraction is used to obtain high gelatin content from the marine organisms [60]. Based on the extraction types, each method has both advantages and disadvantages (Table 3).

### 3.2. Protein Hydrolysis

**Figure 4:**
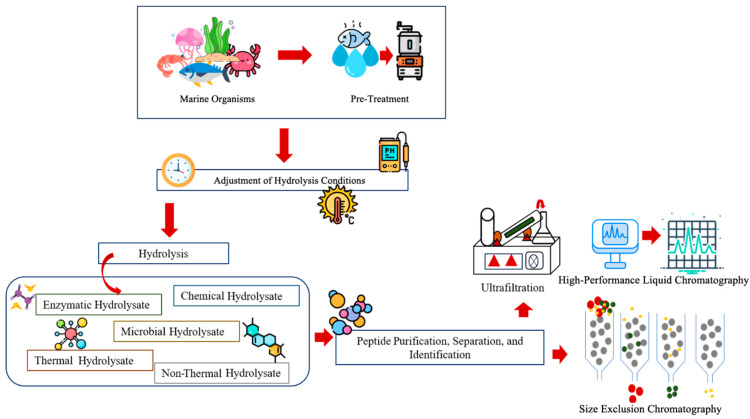
Preparation and identification of marine protein hydrolysates.

Protein hydrolysis is the process of cleaving large protein molecules into low-molecular-weight peptides or individual amino acids, involving a molecule of water for each broken bond [67]. Nowadays, marine organisms and their by-products, especially those derived from fish, are widely recognized as a significant source of proteins, minerals, and fatty acids. On average, the value of fish by-products reaches a content of 49.22–57.92% proteins. Several methods, such as enzymatic, chemical, microbial, thermal, and non-thermal hydrolysic, are involved in the formation of protein hydrolysate [68]. Following protein hydrolysis, products are subjected to purification, separation, and peptide or amino acid identification (Figure 4). Enzyme hydrolysation is a highly efficient method in comparison to other methods, and uses several enzymes such as pepsin, trypsin, neutrase, and protease while maintaining concentration, temperature, and pH [34,69].

Previous research has reported on enzymatic hydrolysis-based bioactivities and functional properties from fish and their byproducts. Still, few studies have focused on the cod backbone as a substrate for continuing this enzymatic hydrolysis. The defrosted cod fish backbones were hydrolyzed using alcalase, neutrase, and protamex at an enzyme-to-substrate ratio of 1% for 24 h under optimal pH conditions with values of 8.0, 8.0, and 6.5, respectively, while the optimal temperatures were 60 °C, 60 °C, and 50 °C. According to the results, the alcalase enzyme-treated cod backbone sample has shown the highest results compared with other enzymes [69]. In another study with olive flounder (*Paralichthys olivaceus*), the researchers aimed to optimize the hydrolysis conditions using their byproducts, resulting in a high-quality fish protein hydrolysate using the alcalase, pepsin, trypsin, protamex, and neutrase enzymes [70]. The amino acid profiles of this research showed high concentrations of Glycine, L-glutamic acid, and L-aspartic acid. Further, researchers are focused on evaluating whether sea cucumber intestinal hydrolysates (SCIHs) can enhance the proliferation and migration of bone marrow mesenchymal stem cells. The research used alkaline protease enzyme to prepare an SCIH [71]. The dried gut and skin of the sea cucumber, *Holothuria scabra*, when mixed with papain enzyme, undergo hydrolysis and contain highly bioactive properties that can have an effective antiproliferative effect on cancer cells. Hydrolysate represents potential as a functional ingredient with natural antioxidant and anticancer properties [72]. In recent years, research has focused on new strategies that can enhance peptide bioactivity through advanced enzymatic hydrolysis techniques, including the use of multiple proteases, membrane–bioreactor systems, and various pretreatment strategies. The results have demonstrated that combining physical pretreatments such as pulsed electric field or high hydrostatic pressure (HHP) with enzymatic hydrolysis can significantly improve protein extraction efficiency, increase the degree of hydrolysis, and generate peptides with stronger bioactive properties, such as antioxidant capacity, which ultimately suppresses inflammation [34].

Chemical hydrolysis is another method that involves the hydrolysis using an acid or base solution to cleave proteins into peptides and individual amino acids. Most commonly, hydrochloric acid (HCl) or sulfuric acid (H_2_SO_4_) for acid hydrolysis, and sodium hydroxide (NaOH) or potassium hydroxide (KOH), are utilized. Compared to other hydrolysis methods, chemical hydrolysis comes with several limitations, such as incompatibility with food applications, lack of control over the hydrolysis process, degradation or destruction of certain amino acids, degradation of tryptophan, need for neutralization, and reduced functional properties due to salt formation [73]. *Fucus vesiculosus* and *Saccharina latissima* are the brown seaweeds that contain glucose and mannitol. Using acidic hydrolysis (0.2 M H_2_SO_4_) and enzymatic hydrolysis (Cellic CTec2 enzyme), one could compare these two types of final results to gain a high yield of glucose and mannitol based on the enzymatic reaction process [74].

*Palmaria palmata* (Dulse) is a red seaweed that contains a limited variety of dietary uses due to ineffective protein extraction methods. Microwave digestion, acid hydrolysis, and Viscozyme hydrolysis are employed for extracting protein from dulse. Ultimately, microwave digestion and acid hydrolysis show high protein amounts and lower-molecular-weight proteins compared to the enzyme hydrolysis method [75]. Microwave hydrolysis offers a sustainable approach to processing seaweeds for protein and nanocellulose management. Using microwave-assisted hydrolysis for *Ascophyllum nodosum* and *Aegagropila linnaei* results in a high yield of proteins, polysaccharides (including alginate and fucoidan), and nanocellulose [76].

### 3.3. Peptide Separation, Purification, Identification, and Quantification Techniques

#### 3.3.1. Ultrafiltration (UF)

Fractionate peptides are based on molecular weight. The membrane separation process is used to purify peptides by selectively retaining large molecules while allowing smaller ones and water to pass through [77]. This ultrafiltration method belongs to the separation and purification of both steps. Membrane fouling is a significant obstacle in industrial UF to the recovery of bioactive peptides, increased energy, and potentially irreversible membrane clogging [78].

#### 3.3.2. Solid Phase Extraction (SPE)

Solid-phase extraction (SPE) is widely used for isolating, purifying, and concentrating peptides from liquid samples using a solid sorbent [77]. This process is also very useful for desalting proteins and sugar samples. SPE resulted in the development of new extraction techniques, such as solid-phase dynamic extraction (SPDE), matrix solid-phase dispersion (MSPD), microextraction by packed sorbent (MEPS), stir-bar sorptive extraction (SBSE), and solid-phase microextraction (SPME) [78,79].

#### 3.3.3. Gel Filtration Chromatography (Size Exclusion)

These separation techniques distinguish molecules based on their size and shape. Gel filtration chromatography is a simple method in which an inert gel medium made up of spherical beads with stable properties is used. When analytes of various sizes are applied, larger molecules pass through the interstitial space and elute first, while smaller molecules diffuse into the pores [77]. Sephadex G-10 is a gel filtration resin used for the desalting and buffer exchange of peptides and small biomolecules with molecular weights over 700. Different types of Sephadex are available, depending on their degree of cross-linking, swelling point, and molecular fractionation range. Sephadex G-10 for small molecules, G-75 for larger molecules, and G-50 are available in the four main categories of particle size: coarse, resin, fine, and superfine. Coarse and resin are preferred for the large scale. Superfine has the smallest bead size for higher efficiency fractionation with a shorter diffusion distance [80]. The collected Sephadex fraction was then analyzed using a UV/VIS spectrophotometer (220 nm), and after that, peptide identification was performed by using HPLC [81]. The molecular mass of the purified peptide and amino acid sequencing is mostly determined by using a Q-TOF mass spectrometer coupled with an electrospray ionization (ESI) [82].

#### 3.3.4. Ion Exchange

Peptides are separated based on their charge. Cation exchange (CM-Sepharose) attaches positively charged peptides. Anion exchange (Diethylaminomethyl (DEAE)-Sepharose) attaches negatively charged peptides. This method is a high-resolution, acid-resistant technique used for separating peptides [83]. It involves a phase with fixed ionic groups and exchangeable ions that can undergo reversible exchange. Purified immunoglobulin G from rabbit serum is obtained using a DEAE-Sepharose fast flow ion exchange column with a Tris-HCl buffer at pH 7.0 and 8.5 as the mobile phase. The separation of proteolytic enzymes with the same net charge and one basic residue is influenced by specific activities. Based on the high resolution, the serum is resistant to acids and alkalis, and can be easily separated into peptides. This purification method can be introduced as a cost-effective marine peptide purification method compared with the RP-HPLC method [37]. Further, using the UV/Vis spectrophotometer in the 200–280 nm range and coupling liquid chromatography–mass spectrometry (LC-MS/MS) provides optimization for both the separation and identification of peptides [81,84].

#### 3.3.5. Reverse-Phase High-Performance Liquid Chromatography (RP-HPLC)

High-resolution purification is based on hydrophobicity. Reversed-phase liquid chromatography (RPLC) purifies proteins based on their polarity. This method introduced a good standard method for the separation, identification, and purification of peptide molecules. This method’s stationary phase functionalizes with an anionic group that can bind positively charged purified peptides [77,85]. Reverse-phase high-performance liquid chromatography (RP-HPLC) is a specific type of RPLC, and this method refers to the same liquid chromatography method. Polar mobile phase and non-polar stationary phase separate compounds based on their hydrophobicity into C18 and C8 columns [86]. Protein identification is based on which proteins are available in the extracted sample. Proteins after the enzymatic digestion were analyzed by mass spectrometry (MS), and the fragment pattern matched the parental proteins. The combination of HPLC and MS can be used to separate and identify peptides [81].

#### 3.3.6. Capillary Electrophoresis (CE)

Bio-capillary electrophoresis is an analytical separation technique that is used to separate peptide samples based on their electrophoretic mobility. In this technique, only a small sample volume and substrates are used. This CE method is more automated and has high resolution compared with other peptide purification methods [87]. Ahn et al. (2015) discussed the involvement of the CE in marine peptide separation in high-throughput and rapid screening of marine protein hydrolysate from *Acetes chinensis*, shark meat, *Polysiphonia urceolata*, *Spirulina platensis*, and mackerel bone, which are enriched in marine peptides with angiotensin I converting enzyme inhibiting activity, checked by using CE [88].

#### 3.3.7. Matrix-Assisted Laser Desorption/Ionization (MALDI) Time-of-Flight (TOF) Mass Spectrometry (MS)

MALDI-TOF MS, or MALDI TOF, is a peptide identification technique. MALDI is a system in which the sample is mixed with a matrix spotted on a stainless-steel plate, where it is evaporated until dry and eventually ionized with a laser. MALDI is often combined with a TOF Mass analyzer, and these methods are effective for analysis with applications in proteomics and food safety [89]. Generally, MALDI-TOF MS determines the molecular weight as 1570 Da, and MALDI-TOF/TOF MS analyzes structures as cyclic peptides [90,91]. The study performed by Panteleev et al. (2020) discussed the structure elucidation and functional studies of a novel hairpin antimicrobial peptide from the marine Polychaeta *Capitella teleta*. The purified peptides were subjected to RP-HPLC for fractionation and finally analyzed by MALDI-TOF MS [90]. Further, identification of bioactive peptides (QTDDNHSNVLWAG-FSR) derived from *Pyropia haitanensis* and converting enzyme inhibitory peptides YRD, AG-GEY, VYRT, VDHY, IKGHY, LKNPG, LDY, LRY, and FEQDWAS from *Palmaria palmata* were investigated by combination with MALDI-TOF and Edman degradation [89,92].

## 4. Anti-Inflammatory Mediators and Their Therapeutic Potentials

### 4.1. Anti-Inflammatory Potential of Marine Bioactive Peptides

**Figure 5:**
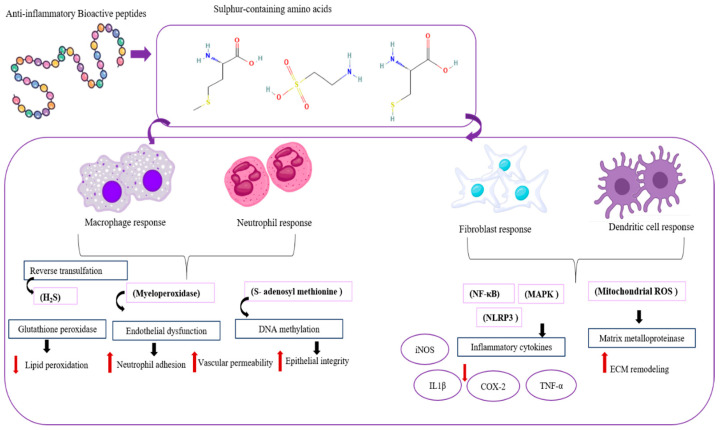
Mechanisms of anti-inflammatory peptides in regulating the inflammatory signaling pathway.

Inflammation is a physiological process that provides an essential defense mechanism for the human body in natural [93]. Often, body tissues and cells are attacked by physical, chemical, and pathogenic hazardous substances; however, acute and chronic diseases are mostly underlined by inflammatory mechanisms of pathogenesis [94]. Primarily, the inflammatory responses can be identified as redness, swelling, heat, pain, and loss of tissue function. Acute (excessive inflammation) and chronic inflammation are the major types of inflammation that can be found in the body. Acute inflammation lasts for short periods and responds by minimizing the approach injury or infection, which helps the reimplantation of tissues. Chronic inflammation lasts for long periods and is mostly not under control, and can develop into chronic inflammation. Recently, excessive inflammation has been discovered as a factor that exacerbates non-communicable diseases (NCDs) such as obesity, diabetes, cancer, respiratory disorders, and cardiovascular disease [95,96]. Marine organisms are a valuable source of proteins and bioactive peptides with medicinal properties. Lectins and phycobiliproteins, like compounds from seaweeds, show biological activity in animal models. These compounds are involved in processes like cell communication, host–pathogen interactions, cancer metastasis, and apoptosis. ESA-2 lectin from *Eucheuma serra* has been shown to induce colonic carcinogenesis when administered orally. The anti-inflammatory mechanisms of MBPs, iNOS, COX2, NFκB, chemokines, cytokines, and MAPK inhibition effects can be studied (Figure 5).

Macrophages, neutrophils, fibroblasts, and dendritic cells all contribute to the anti-inflammatory mechanisms. Macrophages have different phenotypes: M1 and M2. Type M1 promotes inflammation. M2 type secretes cytokines IL-10 and TGF-β, clears apoptotic cells, promotes tissue repair, and prevents chronic inflammation (R1). Neutrophils mostly cause inflammation to resist threats. After being free from harm, apoptosis releases lipoxins and resolvins that balance the immune system and stop inflammation. Fibroblasts assist in restoring tissue integrity by producing extracellular matrix compounds and releasing TGF-β and PGE2, modulating immune cell activity. Dendritic cells regulate immune responses by IL-10 and are induced by regulatory T cells, and suppress excessive inflammation [97]. Inflammation is associated with iNOS proteins. Mainly, free radicals are generated with this iNOS activity. This iNOS produces nitric oxide (NO) from the conversion of L-arginine to L-citrulline. Reactive nitrogen species (RNS) that cause oxidative stress in cells are produced by NO_2_, and NO can produce various types of inflammatory responses [96,98].

### 4.2. Key Mechanisms of the Anti-Inflammatory Mediators

#### 4.2.1. MAPK Inhibition of Marine Organisms

Mitogen-activated protein kinase modules, also known as MAPK or MAP kinase, play a critical role in regulating mammalian cell proliferation, differentiation, and apoptosis [99]. Its primary function is to convert extracellular signals into intracellular responses, facilitating the transformation of stimulus energy into neural activity. The MAPK module consists of three major protein kinases: p38 kinase, c-Jun N-terminal kinase (JNK), and extracellular regulated protein kinase ½ (ERK1/2) [100]. These proteins are activated by various extracellular and intracellular factors, including oxidative stress, osmotic pressure, and pro-inflammatory cytokines. Novel antioxidant peptides (LSPGEL, VYFDR, and PGPTY) derived from the red alga *Gracilariopsis lemaneiformis* were shown to reduce oxidative stress and modulate the inflammatory pathways. According to the results, the isolated peptides were found to suppress excessive NO production and regulate iNOS expression in macrophages [101]. NO is highly involved in monocyte function differentiation and modulates the bioavailability of cells that respond to pro-inflammatory stimuli. The immunomodulatory effects of proper iNOS inhibition in monocytes suggest potential therapeutic strategies for pathological conditions with imbalanced immune responses [102].

Tripeptides from salmon byproducts hydrolysate have shown notable anti-inflammatory properties. These peptides were identified as Pro-Ala-Tyr (PAY) and inhibit NO/iNOS, PGE2/COX-2 pathways, and can also inhibit pro-inflammatory cytokines (TNF-α, IL-6, and IL-1β). *Apostichopus japonicus*- and *Acaudina leucoprocta*-isolated peptides GPSGRP, GPAGPR, PQGETGA, and GFDGPEGPR inactivate MAPK pathways in LPS-induced mouse liver injury [103]. To further understand the anti-inflammatory effects of sea cucumbers, studies have examined mRNA expression in lung tissue related to inflammation signaling. The results indicate that sea cucumber extracts suppressed NF-κB and MAPK transcript levels. This suggests that sea cucumber extracts act as anti-inflammatory agents by inhibiting key mediators of inflammation [100]. Additionally, protein hydrolysates from *Ulva* spp. (green macroalgae) are shown to modulate MAPK pathways, including ERK1/2, JNK, and P38 MAPK. Lymphocytes and macrophages have the potential to regulate the immune response towards their bioactive peptide [104].

#### 4.2.2. NF-κB Inhibition of Marine Organisms

The nuclear factor kappa-light-chain-enhancer of activated B cells (NF-κB) is a transcription factor crucial for both innate and adaptive immune responses, cell growth, and cell development, mainly for inflammation [105]. Further, it regulates more than 500 cancer-related genes [106]. NF-κB activation causes different autoimmune, inflammatory, and malignant disorders. Therefore, inhibition of NF-κB signaling can be used in therapeutic applications [107]. Further, the above innate and adaptive immune responses are activated using the ligation of T or B cell receptors that are involved in various inflammatory diseases. The inhibition of the activation of the NF-κB helps the pathogenic process of various inflammatory diseases. The NF-κB is a central mediator of pro-inflammatory gene induction. *Isostichopus bandionotus* and *Cucumaria frondosa* have strong anti-inflammatory properties [108,109]. The IKK kinase is a central regulator of the NF-κB pathway, composed of two catalytic subunits (IKK α and IKK β) and a regulatory subunit, IKK γ. Phosphorylation of IKK β protein activation, leading to degradation, allows NF-κB dimers to translocate into the nucleus and regulate the transcription of pro-inflammatory and immune-related genes [110]. *Chlorella pyrenoidosa* is a unicellular photosynthetic green alga rich in protein and fats. Chlorella-11 peptide, which contains *C. pyrenoidosa*, inhibited the MCP-1 formation in LPS-stimulated Raw 264.7 macrophages, blocked intercellular adhesion molecule-1 and vascular cell adhesion molecule-1 formation in endothelial cells, and lowered the NF-κB abundance [111]. LDAVNR and MMLDF are two peptides purified from *Spirulina maxima* hydrolysate, found to inhibit cytokine production in endothelial cells, indicating their potential to reduce inflammation [104].

Chemokines are small proteins that contain structurally important cysteine residues, which play a crucial role in organ development, normal physiology, and immune responses. Marine-derived peptides produce anti-inflammatory effects by downregulating, such as COX-2 and iNOS, and modulating cytokine and chemokine signaling. Inhibiting pro-inflammatory molecules such as TNF-α, IL-6, and MCP-1 leads to reduced immune cell infiltration and helps restore immune balance, highlighting their role as immunomodulatory agents [112]. Pacific oysters (*Crassostrea gigas*) extract oyster β-thymosin water-soluble polypeptide. Peptides were tested for anti-inflammatory activity. These peptides significantly inhibited NO production, suppressed PGE2, iNOS, and reduced the COX-2, TNF-α, IL-1β, and IL-6and blocked NF-κB pathways and activation by preventing κB (IκB) degradation in LPS-induced RAW264.7 cells. The studies have exhibited a strong positive correlation of strong in vivo osteogenic activity with COX-2 inhibition, showing the potential for novel drugs for treating bone disorders related to inflammatory processes using sea cucumber extractions. The authors discussed the involvement of the isolated clam worm (*Marphysa sanguinea*) peptide NCWPFQGVPLGFQAPP, which suppresses the NF-κB signaling pathway and lowers the NO and iNOS. According to the authors, the anti-inflammatory activity was assessed based on the relative COX-2 expression compared to the control. C- phy-cocyanin is a dominant pigment protein in *Spirulina platensis* and *Synechococcus* marine bacteria peptides (AILQSYSAGKTK, ALNKTHLIQTK, LLVHAPVK, IPDAHPVK, and VVVLRDGAVQQLGTPR), effectively inhibiting inflammation by reducing the abundance of NO synthase and COX-2, promoting the production of pro-inflammatory cytokines [104].

## 5. Current Trends and Future Directions

The diverse bioactivity properties of MBPs have led to significant scientific and industrial interest in various applications. In the pharmaceutical industry, adequate evidence has been provided on production, purification, optimization, and identification in previous studies. Instead of advantages, considerable drawbacks in the production yield, low purity, and high cost on large scales remain. Further, the short half-life of existing peptides and the loss of stability during processing are the other critical aspects that should be addressed immediately [113,114]. Moreover, MBPs often have a strong, unpleasant marine odor and a bitter taste. Flavor modification of MBPs is essential for oral pharmaceutical products to ensure better adherence to treatment [115]. So far, the potential health benefits have mostly been observed in in vitro and in vivo models, and these technologies must be validated through human clinical trials [37].

Therefore, novel technological approaches and regulatory support systems have led to improvements in the process of peptide isolation, purification, identification, and quantification from various marine biological sources. Sustainable and rapid development of each step allows for novel peptides that could be a solution for health issues. Currently, enzymatic hydrolysis is the main and common method for producing bioactive MBPs rather than conventional methods such as ultrasound, high hydrostatic pressure, microwave, pulsed electric field, and subcritical water. Most of these methods are particularly suitable only for laboratory-scale implementations, and large-scale production is often limited [23]. However, the low yield and high cost of the enzymatic method remain, motivating researchers to seek alternative technologies. Application of bioinformatics, computational approaches such as molecular docking, in silico screening, and artificial intelligence (AI) will lead to a better understanding of metabolic pathways, molecular mechanisms, and target interaction of each bioactivity [116]. AI, particularly deep learning (DI), has emerged as a transformative peptide-based drug-designing tool capable of analyzing complex structural data more precisely. Recent advanced tools such as AF3/AFM and RFAA/RoseTTAFold can predict high-resolution 3D structures of protein and peptide complexes. Furthermore, these models capture complex molecular structures and increase target binding potential more accurately and quickly, making it easier to develop new peptide-based therapeutics [117].

Further, the development of omics technologies such as genomics, transcriptomics, and proteomics has greatly advanced the discovery of novel MBPs with potential health benefits. The rapid development of these technologies has facilitated the discovery of novel peptides, their sequences, and their functionality by increasing yield and minimizing the cost. The molecular hybrid approach can be proposed to enhance the therapeutic effects of the doses by reducing or removing toxicity compared to currently available single-molecule-based treatments [28]. Because of their unique and complex biological mechanisms, these hybrids require careful evaluation, including studies on their toxicity and pharmacokinetics. Modification of the flavor and stability of the peptides is important for developing MBPs. Nanotechnology can significantly enhance the performance of marine peptide-based drugs by overcoming the drawbacks of poor stability, short half-life in the body, and difficulty reaching the target. Nano-encapsulation of marine peptide-based drugs into various structures leads to protecting their functional activity and stability. Nanoliposomes, nanoemulsions, and polymeric or lipid nanoparticles are the most common nanocarriers that are essential to overcome these limitations [118].

## 6. Conclusions

In conclusion, bioactive peptides derived from marine sources are promising biomolecules for treating several diseases due to their diverse bioactivities. For inflammation, several approaches have been explored to combat it so far; however, MBPs allow additional benefits that are still unable to be achieved using conventional treatments, even to treat chronic diseases. Even though MBPs serve as sustainable and highly effective bioactive compounds, it is necessary to accurately identify the relationship between specific amino acid sequences and anti-inflammatory features for various effects, including more reliable targets and mechanisms, controllability, and reproducibility abilities, in order to enhance their powerful anti-inflammatory activities. For this purpose, modifications of many peptide parameters use novel technologies, particularly multi-omics strategies and high-throughput screening methods in biotechnology. Specifically, future studies should be focused on the detailed characterization of proteoforms to better understand the mechanisms underlying the bioactivity of MBPs, as the specific modifications or cleavage patterns in source proteoforms can influence peptide activity. Moreover, clinical studies that are more focused on humans, extending beyond in vitro experiments, are essential. These efforts aim to achieve safe and effective marine peptide-based therapeutics that not only address inflammation but also other life-threatening diseases in the near future.

## Figures and Tables

**Table 1:** The status of marine-derived peptide pharmaceutical products undergoing clinical trials.

Compound	Derivative/Natural Product	Marine Source	Application	Status	Reference
Glembatumumab vedotin	Derivative	Dolastatin 10 from *Dolabella auricularia*	Cancer Treatment	Phase I/II Clinical Study	[38,39,40]
Plitidespin	Natural product	Cyclic Depsipeptide from *Aplidium albicans*	Cancer Treatment	Phase I/II Clinical Study	[41,42]
DU145 (Soblidotin)	Synthetic Derivative	Sea Hare (*Dolabella auricularia*)	Cancer Treatment	Phase I Clinical Study	[43]
Plitidepsin	Derivative	Cyclic Depsipeptide from *Aplidium albicans*	Antiviral (SARS-CoV-2)	Phase III	[3,44]

**Table 2:** The structure of MBPs affects their different functions.

Structural Features	Source of MBPs	Feature Highlighted	Biological Activity	Key Findings	References
MW of the marine peptide	*Lophius piscatorius* (monkfish) swim bladder hydrolysate fraction	<1 kDa	Antioxidant	Scavenging free radicals (51.57 ± 1.45 and 76.96 ± 2.40% for DPPH and HO)	[23,50]
	*Ulva prolifera*	<3 kDa	ACE Inhibitory	High ACE inhibitory activity with low-MW peptides (IC_50_ = 0.036 mg/mL)	[51]
	*Hippocampus abdominalis*	<5 kDa	ACE Inhibitory	High ACE Inhibitory activity with low-MW peptides (IC_50_ = 0.044 mg/mL)	[52]
Amino acid composition and sequence	EVPLFR from *Cucumaria frondosa*, RWDISQPY from *Sargaddum maclurei*, SEGPK, FDGPY, and SPGPW from *Monkfish*	Hydrophobic (Leu, Phe, Trp), aromatic at C-terminus, positively charged (Lys, Arg) in middle, Proline at C-terminal	ACE Inhibitory	Binds to the ACE active site via hydrogen bonding, hydrophobic, and electrostatic interactions	[53,54,55]
	LLVSeMY, MMDSeML (*Oyster*)-GVPLT, GPP, AGLYPGA from fish sources	Aromatic (Tyr, Trp, His), sulfur-containing (Met, Cys), hydrophobic residues	Antioxidant	Scavenges ROS via electron donation (His), H-donation (Cys), or S-atom oxidation (Met)	[55]
	HVLSRAPR from *Spirulina platensis*	Hydrophobic amino acid-charged residues	Anticancer	Improves membrane interaction, enhance selectivity, and reduce cytotoxicity	[23]
Spatial conformation	Turbot viscera hydrolysate from *Scophthalmus maximus*	High α-helical content—strong amphiphilic symmetry	Antimicrobial effect	Enhances membrane disruption and bacterial killing	[56]

**Table 3:** Advantages and disadvantages of protein extraction methods.

Extraction Method	Advantage	Disadvantage	References
Conventional Solvent	Simplicity Practicality Cost-effectiveness	Prolonged extraction times Darkened protein coloration Suboptimal extraction efficiency	[62,63]
Microwave-Assisted	Less use of solvent High extraction efficiency	Filtration step is required Darkened protein coloration	[62,64,65]
High Hydrostatic Pressure	Reduction in microbial load Efficient extraction yield Reduced chemical usage	Limited industrial scalability Potential structural changes	[62]
Pressurized Liquid	Less use of solvent Improved extraction efficiency Excellent compatibility for food-grade purposes	High maintenance cost Potential protein degradation	[62,65]
Pulsed Electric Field	Higher extraction yield Maintains protein integrity Shorter processing time	High equipment cost Higher electricity consumption Limited industrial scalability	[62]
Enzyme-Assisted	Increased yield of extract Non-toxic Non-flammable	High cost of enzyme Difficult to scale up	[62,64,65]
Ultrasound-Assisted	Reduced use of solvent Lower extraction temperatures Shorter extraction time	Possible degradation of heat-sensitive compounds and formation of free radicals	[58,62,66]
Deep Eutectic Solvent Extraction	Numerous combinations Low toxicity Cost-effective	Prolonged extraction time Potential protein degradation	[64]
Physically-Aided Extraction	Increased efficiency Reduced extraction time Intact collagen structure	Limited industrial scalability Difficult steps	[64]
Extrusion–Hydro-Extraction	High yield Reduced waste Continuous production	High equipment cost Filtration step required	[64]

## Data Availability

Not applicable.
